# Supplementary material for: Causal interplay between lactose intolerance and gut microbiota: a combined bidirectional Mendelian randomization and in vivo validation study
Source: Front Nutr. 2026 Jun 1;13:1803337. doi: 10.3389/fnut.2026.1803337 (PMC13265576; doi:10.3389/fnut.2026.1803337)
Supplement: Supplementary file 1 [file Data_Sheet_1.zip › supplementary materials/Forward/leave one out cross validation/ebi-a-GCST90027498.finngen_R12_E4_LACTONAS.pdf]

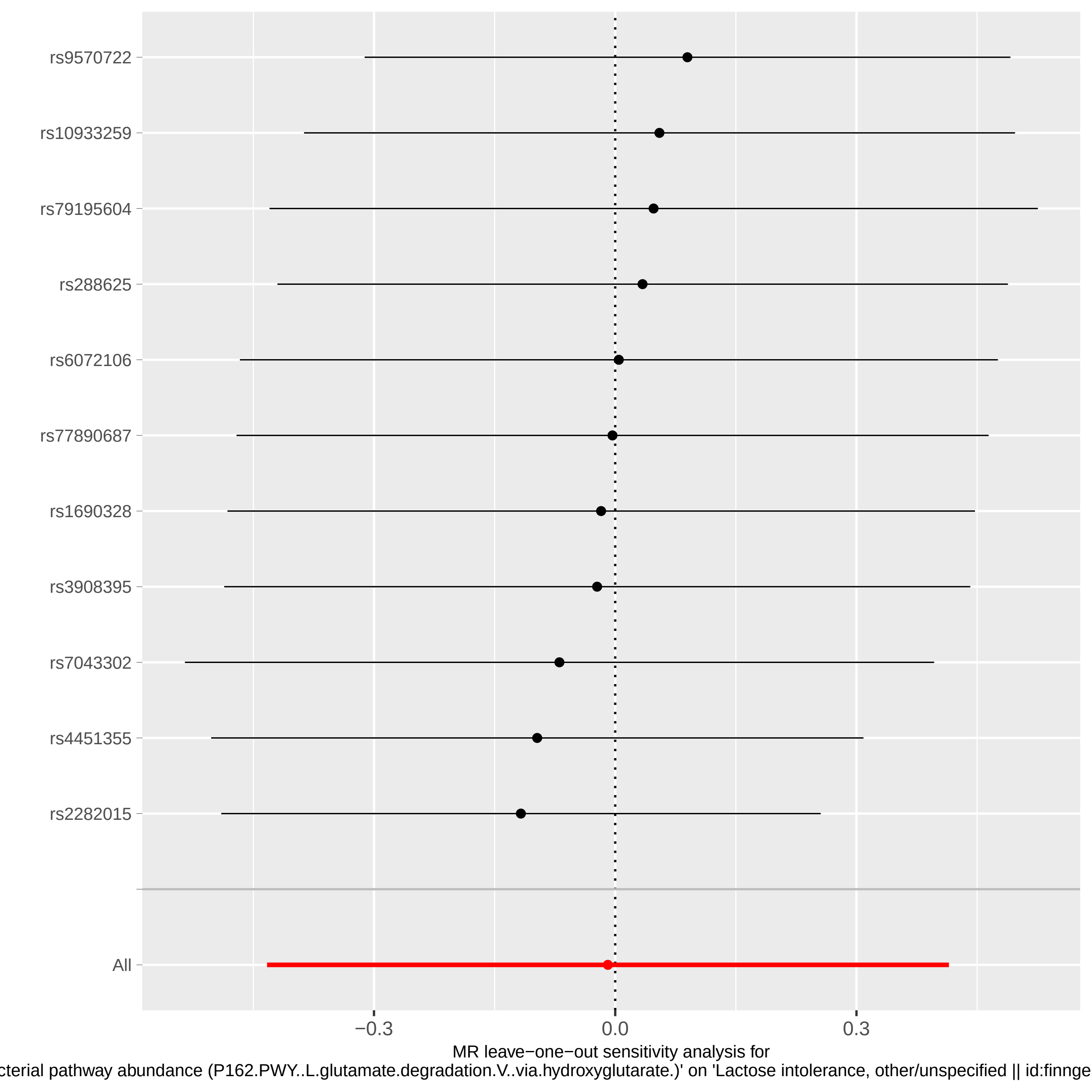
rs9570722
rs10933259
rs79195604
rs288625
rs6072106
rs77890687
rs1690328
rs3908395
rs7043302
rs4451355
rs2282015
All
−0.3
0.0
0.3
MR leave−one−out sensitivity analysis for
cterial pathway abundance (P162.PWY..L.glutamate.degradation.V..via.hydroxyglutarate.)' on 'Lactose intolerance, other/unspecified || id:finnge
